# Nitrogen Use Efficiency in Durum Wheat Under Different Nitrogen and Water Regimes in the Mediterranean Basin

**DOI:** 10.3389/fpls.2020.607226

**Published:** 2021-02-10

**Authors:** Antonio Lupini, Giovanni Preiti, Giuseppe Badagliacca, Maria Rosa Abenavoli, Francesco Sunseri, Michele Monti, Monica Bacchi

**Affiliations:** Dipartimento di Agraria, Università Mediterranea di Reggio Calabria, Reggio Calabria, Italy

**Keywords:** *Triticum turgidum* spp. *durum*, water use efficiency (wue), heritability, nitrogen uptake efficiency (NUpE), nitrogen utilization efficiency (NUtE), post anthesis nitrogen uptake (PANU)

## Abstract

Improving nitrogen use efficiency (NUE) represents one of the main goals to reduce N input in maximizing crop yield for sustainable agriculture. A NUE key strategy is the exploitation of genetic variation in available germplasm together with the understanding of molecular mechanisms governing this complex trait. Thus, NUE, its components, nitrogen uptake efficiency (NUpE) and nitrogen utilization efficiency (NUtE), and NUE-related traits heritability were evaluated in ancient (Cappelli, Capeiti, Russello, and Mazzancoio) and modern (Messapia, Tiziana, Svevo, and Normanno) wheat genotypes for tackling nitrogen (N) and/or water limitation in both growth chamber and field experiments. Our results exhibited a reduction of NUE, NUpE, and NUtE under water and combined (nitrogen + water) stress in all the genotypes, as expected. The contribution of genetic variability on phenotypic variation was significant for NUtE, harvest index, post-anthesis nitrogen uptake (PANU), and biomass production traits. Moreover, the stress tolerance indexes, calculated and bi-plotted for N and water stresses, exhibited two distinct clusters for many traits as then confirmed by principal component analysis. Although modern varieties showed higher crop yield and NUE under conventional N and water regimes, ancient varieties exhibited best performances to cope with both stresses, mainly under water limitation. Finally, the usage index, which takes into account total biomass increase, underlined that old genotypes were less affected by both stresses during crop cycle. In particular, these genotypes showed the best performances for NUE and its components under both stresses at stem elongation and milk ripening as shown also by PANU. In addition, at these stages, nitrate and ammonium transporter gene expressions in the root were performed, showing the highest activity in ancient varieties. In conclusion, the identification of NUE traits during a specific crop cycle stage, under both N and water limitation, will help in the breeding of more resilient varieties in Mediterranean sustainable agriculture by reducing N supply.

## Introduction

Fifty percent of total human calories are supported by cereal crops, and durum wheat (*Triticum turgidum* L. subsp. *durum*) is among the most important crops in the Mediterranean basin (Padovan et al., 2020). Moreover, global demand for food, feedstock, and biofuel due to the growing world population is expected to double by 2050; therefore, improving crop yield for tackling the future demand is the main challenge in the next decades (Rathore et al., 2017). Otherwise, to underpin sustainability in cropping systems, improved resource utilization should be achieved to reduce fertilizer input. Moreover, another strategy for increasing crop yield may be the utilization of marginal areas, where natural resources are limited. In both cases, the selection of genotypes with higher resource use efficiency should be the main target in the near future. In this context, nitrogen and water availability, respectively, represent the main constraints limiting crop yield (Passioura, 2002). Indeed nitrogen may be considered as the driving force for plant development, thereby balancing other nutrients (Hawkesford and Griffiths, 2019). Recently, the massive use of N fertilizers determined significant harmful effects on biodiversity and the functioning of terrestrial and water ecosystems, air pollution as well as human health (Hirel et al., 2007), with only 33% really used by plants (Raun and Johnson, 1999). In addition, a robust relationship between nitrogen utilization by crops and water stress, in which optimal N nutrition can ensure normal metabolic processes even under water stress conditions, has been reported (Zhang et al., 2007; Ata-Ul-Karim et al., 2016). The interactions between water and nitrogen use efficiency (WUE and NUE, respectively) were also highlighted in wheat when considering leaf photosynthetic gas exchange (Wang et al., 2016).

In contrast, water deficiency reduces grain yield, nitrogen uptake, and evapotranspiration, which can be restored by water supply, by reducing water use efficiency (Plaut et al., 2004; Sun et al., 2006; Zhang et al., 2006).

To develop sustainable cropping systems by reducing environmental and health costs and sustaining high crop yields (Passioura, 2002; Good et al., 2004; Hirel et al., 2007; Sebilo et al., 2013), management strategies toward lowering irrigation and N fertilizer rates must be adopted (Quemada and Gabriel, 2016). WUE and NUE would become achievable goals by using novel resilient genotypes more capable to uptake and utilize water and N available in the soil (Fleury et al., 2010; McAllister et al., 2012).

The NUE and WUE concepts imply a complex framework of multiple physiological processes involved in a plant’s ability to efficiently uptake and utilize N and water inputs. Several definitions and methods for measuring both NUE and WUE have been developed over the years (Good et al., 2004; Sadras, 2004). NUE was defined as the grain yield per unit of N available from the soil, including N fertilizer (Moll et al., 1982), and also as the fresh matter (FM) or dry matter (DM) produced per N content or per N unit taken up from the soil, mainly for biomass production (Good et al., 2004; Arregui and Quemada, 2008). Besides this, WUE was defined as crop yield or biomass per water consumed calculated as evapotranspiration (Tanner and Sinclair, 1983; Sadras, 2004), which is useful to compare different agronomic management practices (Quemada and Gabriel, 2016). Moreover, due to their complexity, simulation models have been developed to study the WUE and NUE interaction, taking into account different variables (soil, rainfall, and so on) to confirm field experiments (Asseng et al., 2001).

Indeed NUE is a complex trait controlled by interacting genetic and environmental factors. Two physiological components were defined: nitrogen uptake and utilization efficiency (NUpE and NUtE, respectively). The first describes a plant’s ability to take up N from the soil; the second one refers to the ability of a plant to convert in biomass the assimilated/remobilized nitrogen (Good et al., 2004; Xu et al., 2012; Abenavoli et al., 2016; Mauceri et al., 2020). Therefore, to understand the different strategies in N acquisition, other NUE definitions were utilized in different contexts such as N physiological efficiency (NpUE), N recovery efficiency (NRF), apparent nitrogen recovery rate (ANR), agronomy efficiency of N fertilizer (AE), and N remobilization efficiency (NRE) (Xu et al., 2012; Han et al., 2015; Quemada and Gabriel, 2016). Since the first NUE network step is N uptake from the soil, the transport proteins are key targets for improving N efficiency. Many transporters are encoded in plants to respond to different N forms in the soil as nitrate (NO_3_^–^) and ammonium (NH_4_^+^), which represent the major forms of nitrogen (N) uptake in higher plants. Nitrate concentration in the soil is highly variable due to fluctuation, and its uptake is governed by at least two transport systems, low- and high-affinity transport systems (LATS and HATS—operating at high and low N concentrations, respectively). In particular, HATS allow plants to maximize nitrate acquisition under low N availability or when limited or no N fertilizer is applied (Malagoli et al., 2004). In bread wheat (*Triticum aestivum* L.), a *NRT2.1* transporter belonging to the HATS gene family was isolated and characterized, and its transcript abundances decreased in the roots in response to NO_3_^–^ and NH_4_^+^ (Wang et al., 2011). Unlike nitrate, NH_4_^+^ is commonly buffered by negatively charged soil particles. NH_4_^+^ accumulation in cells occurs either by direct uptake from the rhizosphere *via* ammonium transporters (AMTs) or by reduction of NO_3_^–^. Then, it is assimilated into glutamate *via* the glutamine synthetase/glutamate synthase cycle (Masclaux-Daubresse et al., 2010). Saturable and non-saturable systems operating at low and high external ammonium concentrations were characterized in several plant species. At low concentration, NH_4_^+^ uptake is mediated by AMT1-type transporters (von Wittgenstein et al., 2014).

In the last decades, many attempts were made to identify novel genes involved in N uptake, assimilation, translocation, recycling, and remobilization to increase NUE in crops (Kant et al., 2011; Xu et al., 2012). For this aim, there is potential to utilize genetic variability from old genotypes for developing new cultivars with increased ability to use N.

Indeed the research efforts were addressed to the selection of high NUE genotypes by using allelic variation for NUE traits through phenotyping segregant populations, mapping quantitative trait loci (QTLs), and selecting candidate genes for NUE improvement (Han et al., 2015). Afterward, segregant recombinant inbred line populations were adopted for QTL mapping of traits related to NUE components and yield potential (Xu et al., 2012; Han et al., 2015). Recently, they have been identified in *Arabidopsis*, barley, maize, rice, and wheat mapping populations (Yang et al., 2017; Zhang et al., 2019; Ertiro et al., 2020). Improving NUE should also take into account diverse gene pools; thus, the use of ancient germplasm may represent a useful resource for breeding programs.

The current study was focused on the responses of four modern and four ancient durum wheat (*Triticum turgidum* spp. *durum*) genotypes to limited N and water supply, considering plant growth, yield, and its components and adopting a two-factor experimental design in both growth chamber and field condition. To analyze the wheat responses to both stresses agronomic, physiological and molecular approaches were adopted.

The combination of N and water stress has been recently reported (Islam et al., 2021) and, in our dryland condition, may be useful to identify genotypes within our panel that are more able to maintain NUE performance under water stress. This approach could also highlight tolerance mechanisms in the ancient genotypes included in our study, providing valuable genotype for improving NUE under a Mediterranean environment. This is one of the first reports on NUE and WUE performances and their interaction in different durum wheat genotypes growing under rainfed Mediterranean conditions.

## Materials and Methods

### Plant Materials

Eight durum wheat (*T. turgidum* spp. *durum*) genotypes (Supplementary Table S1) responsive to N and water stresses were evaluated in two different trials: growth chamber pot and field experiments. Four modern varieties, namely, Messapia, Normanno, Svevo, and Tiziana, widely spread during the 1980s and 1990s were included in group 1 (G1). Two ancient varieties, namely, Cappelli and Capeiti, widely cropped during the 1960s and 1970s in South Italy, as well as two old landraces, namely, Russello and Mazzancoio, from Sicily and Calabria, respectively, were included in group 2 (G2). The four modern varieties were characterized by dwarf/small size and earliness, while the four ancient genotypes were characterized by tall size/standard and late maturity.

### Growth Chamber Pot Experiment

In the growth chamber, eight genotypes, two water (W20 and W40) and nitrogen (N0 and N80) levels, were combined and arranged in a completely randomized factorial design with three replications. The seeds were sown in plastic pots (15 × 15 × 30 cm, 0.67 L), filled with 600 g soil (8% humidity) from the experimental station of the Department AGRARIA, and sieved with a 2 × 2-mm wire mesh to remove the coarser part. In both treatments (N0 and N80), 0.68% (w/v) solution of calcium hydrogen phosphate (CaHPO_4_) equivalent to 0.48 g/L of PO_4_^3–^ was distributed in each pot before sowing, and 48 mg KNO_3_ was added 10 days after emergence. At the same time, 35.5 mg KCl was supplied to N0 for balancing K^+^ with the fertilized treatment (N80). The pots were transferred to a growth chamber at 18°C, and at 5 days after emergence, the temperature was raised to 20°C for 20 days using 10/14-h light/dark photoperiod and 340 μmol m^–2^ s^–1^ light intensity. During the remaining 20 days, i.e., until the end of the experiment, temperature and photoperiod were set at 25°C and 14/10 h, respectively. The W20 and W40 treatments were obtained by maintaining 20 and 40% humidity (water available) in the pots, periodically measured by Campbell Scientific’s TDR 100 (Soil moisture TDR technology, Trase) during plant development in the growth chamber.

Three replications (each consisting of five plants) for treatment were collected and divided into shoot and root and then oven-dried at 70°C for 48 h to determine shoot dry weight (SDW, g) and root dry weight (RDW, g). Total nitrogen content (Nc, mg N) was determined by the Kjeldahl method.

Nitrogen use efficiency [NUE, SDW N%^–1^, where N% is the g N (100 g DW)^–1^] (Chardon et al., 2010) and nitrogen utilization efficiency (NUtE, SDW^2^ Nc^–1^) (Siddiqi and Glass, 1981) were calculated. Nitrogen uptake efficiency (NUpE) was also estimated as total (shoot + root) dry weight (TDW) × N concentration (g N g TDW^–1^) (Chardon et al., 2010). The mean of five sampled plants was considered.

### Field Experiment

The same varieties (V) utilized in the growth chamber were evaluated at two N and water levels in the field. The experiment was carried out during the growing season of 2017/2018 at the experimental station of the University Mediterranea of Reggio Calabria, located in Gallina of Reggio Calabria (38°10′ N, 15°45′ E, 232 m a.s.l.). The “Typic Haploxeralfs” (USDA) soil with the following physical–chemical features (0–30-cm depth) was utilized: 35% clay, 25% silt, and 40% sand, pH 7.05, organic matter content 1.75%, total N (Kjeldahl) 1.02‰, P (Olsen) 12.24 ppm, and K 382.18 ppm. The water content at field capacity and wilting point was 30.3 and 17.2% humidity (dry weight), respectively.

Soil analysis was performed to determine the nitrogen mineral forms and total carbon content (Lambda FIAS UV/VIS Spectrophotometer Perkin Elmer): dry weight was measured after drying in an oven at 105°C until a constant mass, and ammonium (NH_4_^+^–N) and nitrate nitrogen (NO_3_^–^–N) (after extraction in 2 M KCl) were determined.

Temperature and rainfall during the wheat growing season were comparable to the 25-year mean of the experimental site. The average monthly minimum and maximum temperature (October–June period) was 10.0 and 23.7°C, respectively. February was the coldest month, and the minimum air temperature dropped to 6.8°C. Air temperature started to increase from March, but frequent variations during March and April and finally a sharp decline in the third decade of May were observed. The total rainfall amounted to 496 mm (the 25-year average in the same period is 552 mm), with the wettest months being November (105.6 mm) and February (103.6 mm) (Supplementary Figure S1).

In a split–split–plot experimental design with three replications, V, W, and N factors were arranged. Two water levels (W and W_*S*_—normal condition and water stress, respectively) were assigned to the main plot, two N availability (N100 and N0: N-normal and N-limiting condition, respectively) to the subplot, and the eight genotypes to the sub-subplot (Supplementary Table S1).

Field trial was carried out in succession to a vetch/oat intercrop for forage. The soil was prepared by summer plowing at 30-cm depth, followed by two harrowing in autumn. Wheat was sown on 27 November 2017, adopting 350 plants per square meter of sowing density. Each plot area was 3.6 m^2^ (six rows at 0.20 m apart). In the N100 subplots, 36 kg ha^–1^ of N and 92 kg ha^–1^ of P_2_O_5_ as diammonium phosphate [(NH_4_)_2_HPO_4_] were added before sowing; in N0, only phosphorus was replenished by supplying 92 kg ha^–1^ of P_2_O_5_ as mineral superphosphate. At 4 weeks after the plants’ emergence, 1-m^2^ areas were identified for destructive plant samplings. To induce *W*_*S*_, eaves gutters (18 cm in width) were placed in 21 January (54 days after sowing) in the inter-row, thus obtaining 90% of rainfall intercepting main plot surface. The water intercepted was conveyed into a ditching system with an adequate slope.

At sowing and during the cropping season, samples were collected from each main plot (W and Ws) at two soil layers, 0–30 and 30–60 cm, respectively, to calculate, according to the gravimetric method, the soil water content variations, with a significant reduction (46%) in the Ws subplot compared to the control (W).

At the end of tillering (February 26), the eaves gutters were removed from the main plot, and in the N100 subplots by N top-dressing application, 64 kg urea was supplied. Weed control was carried out in March 10 by using pinoxaden (Axial60), clopiralid, florasulam, and fluroxipir meptil mixture (Columbus).

At each stage, tillering (TI), stem extension (SE), anthesis (AN), milk ripening (MR), and harvesting (H), five plants were collected from the sampling area to determine total dry matter. The plants were cut at 5 cm from the ground; the leaves and stems were separated, oven dried at 65°C, and weighted to determine their dry matter.

At maturity, above-ground biomass (AGB), plant height (PH), spike number (SN), 1,000 seeds weight (1000SW), and grain yield (GY) were determined from 1-m^2^ sampling area of each sub-subplot. GY was converted into kilogram per hectare at 13% humidity. The nitrogen content of the dry matter was determined by the Kjeldahl method.

The total dry matter and the respective nitrogen content were utilized to calculate nitrogen plant accumulation and partitioning into the spike and grain.

Nitrogen use efficiency and its components (NUpE and NUtE) were calculated according to Moll et al. (1982), while the usage index (UI), over the biological cycle, was estimated according to Siddiqi and Glass (1981). The following N-related physiological parameters were also calculated: Harvest Index (HI; ratio between grain weight and total dry weight), nitrogen harvest index (NHI; N grain content divided by N total in the plant), post-anthesis nitrogen uptake (PANU; total N at harvest minus total N at heading), and NRE (N remobilization divided by total N at anthesis) (Bogard et al., 2010). The AE and physiological efficiency (PE) were estimated as the differences between grain weight with fertilized and unfertilized control for N applied and taken up, respectively (Good et al., 2004).

### Stress Tolerance Index

Stress tolerance indexes (STI-W and STI-N) were calculated for both N and water treatments and each genotype using the following formula (Fernandez, 1992):

$$S\,T\,I=\frac{G_{n}\times G_{S}}{G_{n\,m}^{2}}$$

where *G*_*n*_ and *G*_*s*_ are the yield genotypes under no-stress and stress condition, respectively, and *G*_*nm*_ was the average yield of all the genotypes under no-stress condition.

### Water Use Efficiency

Water used (WU) by the crop in the 0–60-cm layer soil for each treatment was estimated by adding the amount of rainfall and the variation in soil moisture content (Δ*U*), for each time, net of percolation losses. WUE was calculated as the ratio between final grain yield and WU amount (Loss et al., 1997).

### Gene Expression

To evaluate the expression levels in the root of some N-related genes in response to N and W stress, alone or in combination, real-time PCR analysis was carried out at SE and MR stages. Three biological replicates were sampled for each treatment (4) and each genotype (8). Each replicate consisted in a pool of root from six plants. The primers used for each gene target are reported in Supplementary Table S2 according to Saia et al. (2015).

RNA was extracted using RNeasy Plant Mini Kit (Qiagen, Milano, Italy) according to the manufacturer’s protocol, and its quality and quantification were assayed using NanoDrop 2000 (Thermo Scientific). A first-strand cDNA was synthesized from 2 μg of total RNA (Tetro cDNA synthesis kit) using oligo-dT primers as suggested by the Bioline manufacturer. Real-time PCR (qPCR) was performed on DNA Engine Opticon2 (Bio Rad) using SYBR Green master mix kit (Sigma-Aldrich) according to the manufacturer’s instructions. The qPCR was carried out starting from 2 min at 95°C (initial denaturation) and then for 40 cycles consisting of 30 s at 94°C, 30 s at 60°C, and 1 min at 72°C. The qPCR results were analyzed by the 2^–Δ*Ct*^ comparative method as previously described (BioRad Real-Time PCR Application guide) (Livak and Schmittgen, 2001). Relative changes in expression were determined by calculating the ΔCt referring to housekeeping (Ct 18S) genes (Supplementary Table S2).

### Statistical Analysis

The data from both experiments were checked for normality (Kolmogorov–Smirnov test) and tested for homogeneity of variance (Leven median test). Then, the data from the growth chamber pot experiment were analyzed by two-way ANOVA (with variety and stress as main factors), and the means were separated by Tukey’s honest significant difference test (*p* < 0.05) by using the Systat software (Systat Software Inc., Chicago, IL, United States). The data from the field experiment were analyzed as split–split–plot ANOVA by using agricolae package in R software (R Core Team, 2017).

The relationship between NUE and WUE, through regression analysis, was studied. Finally, Pearson’s correlations among morpho-physiological traits were performed by using corrplot package (Wei and Simko, 2017), whereas principal component analysis (PCA) was performed by using factoextra package based on ggplot2 package (Wickham, 2009), both in R software v.3.4.3 (R Core Team, 2017).

## Results

### Growth Chamber Pot Experiment

**FIGURE 1:**
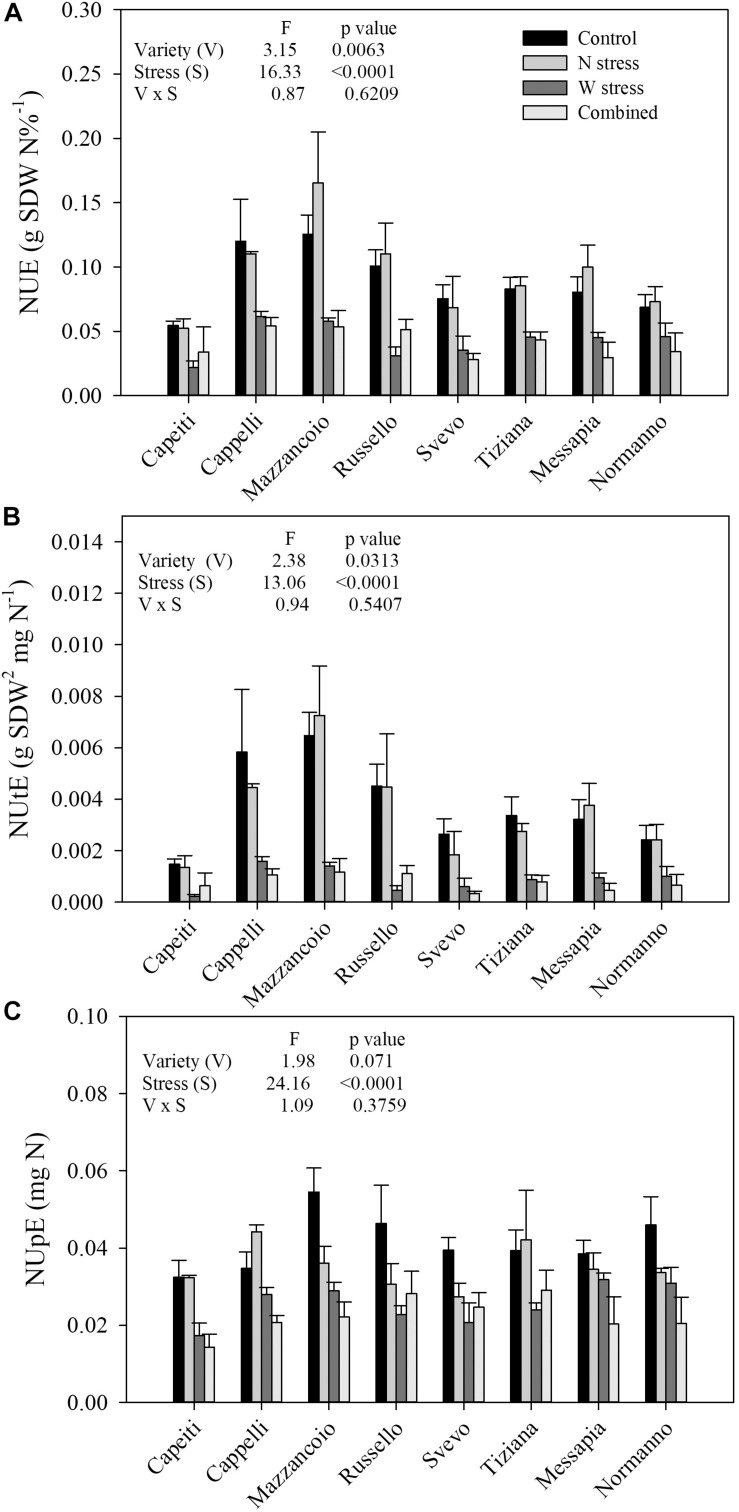
Single and combined effect of N and water stress on nitrogen use efficiency **(A)**, nitrogen utilization efficiency **(B)**, and nitrogen uptake efficiency **(C)** of eight durum wheat varieties grown in a controlled environment. Values are means (*n* = 5) ± SE.

In a controlled condition, the NUE, NUpE, and NUtE of eight durum wheat varieties in response to N and W stress, alone or in combination, were estimated. Variety (V) and stress (S) as main factors were highly significant in all the traits but not its interaction (Figure 1).

At the control (N100/W), NUE ranged from 0.125 (Mazzancoio) to 0.054 (Capeiti), with an average of 0.088 and 28% of variability (coefficient of variability, CV). Furthermore, NUE did not show any significant difference within genotypes when N stress was applied, exhibiting a weak increase in Mazzancoio, Messapia, and Russello. In contrast, water stress significantly reduced NUE in all the genotypes, ranging from 69% (Russello) to 33% (Normanno), with an average of 51% compared to the control, suggesting their different degree of tolerance to drought stress (Figure 1A).

Similar responses were observed for NUtE, where V and S showed significant differences (*p* = 0.0313 and *p* < 0.0001, respectively), unlike V × S (Figure 1B). At the control, a marked NUtE variability among genotypes was detected (CV = 46%), ranging from 0.0064 (Mazzancoio) to 0.0014 (Capeiti) and with an average of 0.0037. As already observed for NUE, N stress did not determine any significant differences among varieties, whereas a marked reduction was induced by water and combined stresses (Figure 1B). In particular, W stress reduced NUtE differently, ranging from 89% (Russello) to 58% (Normanno) and with an average of 75%. Combined stress likewise reduced NUtE, ranging from 87% (Svevo) to 73% (Normanno) (Figure 1B), suggesting a possible additive stress effect.

Finally, although only the S factor was significantly different (*p* < 0.0001) for NUpE, at the control, the genotype responses were quite different, ranging from 0.054 (Mazzancoio) to 0.032 (Capeiti) and with an average of 0.041. Moreover, N stress reduced NUpE in Normanno (26%), Mazzancoio (33%), Russello (34%), and Svevo (30%), whereas an increase was observed in Cappelli (27%). Finally, all the genotypes exhibited a more marked reduction in NUpE when water stress as well as combined stress were applied (Figure 1C).

### Field Experiments

#### Biomass Accumulation and Crop Yield

The biomass accumulation under W and/or N stress during durum wheat biological cycle was evaluated (Table 1). At TI, biomass production was affected by water, N stress as well as their interaction, with Mazzancoio and Cappelli exhibiting the most contrasting performance (327 *vs.* 153 g m^–2^). Similar effects were observed at SE, in which both W and N stress negatively affected the above-ground biomass production in Mazzancoio and Cappelli by 24 and 42%, respectively. At this stage, the highest performance was observed in Messapia (594 g), whereas Capeiti (380.26 g) recorded the lowest value (Table 1).

**TABLE 1:** Agronomic traits of wheat varieties under two N (0 and 100) and water (20 and 40) regimes in field condition.

Factor		DWTI	DWSE	DWAN	DWMR	DWH	ER	1000SW	SN	GY
Water stress	W0	284.70^a^	501.59^a^	965.15^a^	1,370.53^a^	1,459.15^a^	20.56^a^	49.26^a^	242.45^a^	438.07^a^
	Ws	184.95^b^	380.08_*b*_	945.28^a^	1,424.19^a^	1,336.07^a^	20.31^a^	45.24^b^	215.87^a^	370.23^b^
N stress	N0	155.73^a^	324.20^a^	750.69^a^	1,091.23^a^	1,183.10^a^	19.39^a^	49.17^a^	245.25^a^	345.70^a^
	N100	313.93^b^	557.46^b^	1,159.75^b^	1,703.5^b^	1,612.12^b^	21.48^b^	45.33^b^	213.08^b^	462.60^b^
Variety	Capeiti	170.25^de^	380.26^e^	742.92^de^	1,006.53^e^	981.68^d^	16.83^f^	45.92^b^	167^d^	381.21^c^
	Cappelli	152.92^e^	407.93^cd^	710.08^e^	1,657.9^a^	1,589.75^ab^	24.00^c^	49.07^a^	221^bc^	348.88^d^
	Mazzancoio	326.67^a^	429.02^c^	1,367.61^a^	1,701.1^a^	1,601.86^ab^	33.58^a^	48.71^a^	202^c^	367.05^cd^
	Russello	248.76^c^	384.43^e^	1,146.30^b^	1,522.89^ab^	1,637.42^a^	30.33^b^	47.54^ab^	259.66^a^	368.10^cd^
	Svevo	257.37^bc^	533.64^b^	864.95^cd^	1,280.64^cd^	1,238.29^c^	8.08^g^	46.37^b^	250.33^a^	422.79^b^
	Tiziana	276.74^b^	397.63^de^	904.42^c^	1,384.7^bc^	1,459.31^ab^	22.58^d^	48.61^a^	238.66^ab^	445.40^ab^
	Messapia	264.39^bc^	593.97^a^	1,097.59^b^	1,445.9^bc^	1,421.24^bc^	8.17^g^	45.64^b^	237.66^ab^	452.77^a^
	Normanno	181.52^d^	399.97^de^	807.88^cde^	1,179.1^de^	1,251.30^c^	19.92^e^	46.13^b^	257^a^	446.39^ab^
Block		ns	ns	ns	ns	ns	ns	ns	ns	ns
Water (W)		*	*	ns	ns	ns	ns	*	ns	**
Nitrate (N)		***	***	**	***	***	***	*	*	***
W * N		*	**	ns	ns	ns	*	ns	ns	**
Variety (V)		***	***	***	***	***	***	***	***	***
V * W		***	***	**	***	ns	ns	ns	***	***
V * N		***	***	***	*	***	ns	*	***	***
V * W * N		***	***	ns	ns	*	ns	ns	ns	ns

DWTI, dry weight tillering; DWSE, dry weight stem extension; DWAN, dry weight at anthesis; DWMR, dry weight at milk ripening; DWH, dry weight harvest; ER, earliness; 1000SW, thousand seed weight; SN, spike number; GY, grain yield. Different letters along column and within each factor indicate significant differences (least significant difference at *p* < 0.05), whereas *, **, ***, indicate significant differences at p < 0.05, 0.01, and 0.001 respectively.

At AN, MR, and H, significant variations in biomass production were observed only under N stress. In detail, significant differences at AN and MR on above-ground biomass production were found among varieties, ranging from 1,368 g (Mazzancoio) to 710 g (Cappelli) and from 1,701 g (Mazzancoio) to 1,179 g (Normanno), respectively. Finally, at H, only N0 significantly affected biomass accumulation compared to the control (N100), ranking from 1,637 g (Russello) to 982 g (Capeiti) (Table 1).

The earliness was affected by N stress with a significant reduction (10%), unlike W stress, with a range from 33.58 (Mazzancoio) to 8.08 (Svevo) days. Both W and N stress significantly reduced thousand seed weight (1000SW) by 8.1 and 7.8%, respectively. Furthermore, significant differences among genotypes were observed: Mazzancoio, Cappelli, and Tiziana exhibited higher values. Significant differences under N stress among varieties in SN were detected, ranging from 259.6 (Russello) to 167 (Capeiti) (Table 1). Moreover, all the factors (W, N, and V) and their interactions were statistically significant when considering GY. In particular, water and N stress reduced GY by 14 and 25.3% compared to the control, respectively; significant differences for this trait were also observed among genotypes, ranging from 452.77 g (Messapia) to 348.88 g (Cappelli) (Table 1).

HI, NHI, PANU, and NRE did not show any significant variations under water stress (Table 2). In addition, HI was also unaffected by N stress, although a significant variation was observed among varieties, ranging from 0.419 (Capeiti) to 0.230 (Cappelli). Moreover, N stress reduced NHI, showing a significant variation among varieties in which Svevo (0.76) and Mazzancoio (0.61) exhibited contrasting performance (Table 2).

**TABLE 2:** Physiologic traits of wheat varieties under two N (0 and 100) and water (20 and 40) regimes in field condition.

Factor		HI	NHI	PANU	NRE	NUE	NUpE	NUtE
Water stress	W	0.313^a^	0.70^a^	13.87^a^	0.221^a^	7.64^a^	0.65°	12.10^a^
	Ws	0.312^a^	0.69^a^	12.30^a^	0.190^a^	6.97^b^	0.63°	10.99^b^
N stress	N100	0.318^a^	0.72^a^	11.03^a^	0.234^a^	11.08^a^	0.99^a^	11.29^a^
	N0	0.307^a^	0.67^b^	15.14^b^	0.177^b^	3.52^b^	0.30^b^	11.81^b^
	Capeiti	0.419^a^	0.74^ab^	13.75^bc^	0.151^bc^	7.207^bcd^	0.673^a^	10.82^cd^
Variety	Cappelli	0.230^d^	0.70^b^	14.18^ab^	0.204^ab^	6.48^d^	0.631^bc^	10.29^d^
	Mazzancoio	0.234^d^	0.61^c^	15.46^ab^	0.140^c^	6.72^cd^	0.666^ab^	10.04^d^
	Russello	0.231^d^	0.63^c^	15.95^a^	0.138^c^	6.76^cd^	0.649^abc^	10.22^d^
	Svevo	0.357^bc^	0.76^a^	12.04^cd^	0.246^a^	7.431^abc^	0.664^ab^	11.76^bc^
	Tiziana	0.332^bc^	0.71^b^	11.52^d^	0.26^a^	7.845^ab^	0.612^c^	12.89^ab^
	Messapia	0.325^c^	0.73^ab^	11.29^d^	0.255^a^	8.057^a^	0.650^abc^	13.25^a^
	Normanno	0.372^b^	0.70^b^	10.51^d^	0.252^a^	7.967^ab^	0.623^c^	13.13^a^
Block		ns	ns	ns	ns	ns	*	**
Water (W)		ns	ns	ns	ns	*	ns	**
Nitrate (N)		ns	**	**	*	***	***	ns
W * N		ns	ns	ns	ns	ns	ns	ns
Variety (V)		***	***	***	***	***	*	***
V * W		**	***	ns	ns	***	ns	*
V * N		***	**	***	***	ns	***	*
V * W * N		**	***	ns	ns	**	**	**

HI, harvest index; NHI, nitrogen harvest index; PANU, post-anthesis nitrogen uptake; NRE, nitrogen remobilization efficiency; NUE, nitrogen use efficiency; NUpE, nitrogen uptake efficiency; NUtE, nitrogen utilization efficiency. Different letters along column within each factor indicates significant differences (least significant difference at *p* < 0.05), whereas *, **, ***, indicate significant differences at p < 0.05, 0.01, and 0.001 respectively.

Under N stress, PANU was reduced by 27.1%. Significant differences (*p* < 0.001) were also observed among varieties, in which Mazzancoio, Russello, and Cappelli showed higher PANU (15.95, 15.46, and 14.18, respectively) compared to the others (Table 2).

Finally, the NUE and NUtE appeared significantly influenced by both stresses, and the highest values were recorded in Messapia, whereas the other varieties displayed contrasting responses. In contrast, NUpE was significantly affected only by N stress (Table 2).

#### Agronomic and Physiological Efficiency

The agronomic and physiological efficiency (AE and PE, respectively) that already include the responses to N stress were estimated considering the W regimes (W and Ws).

**FIGURE 2:**
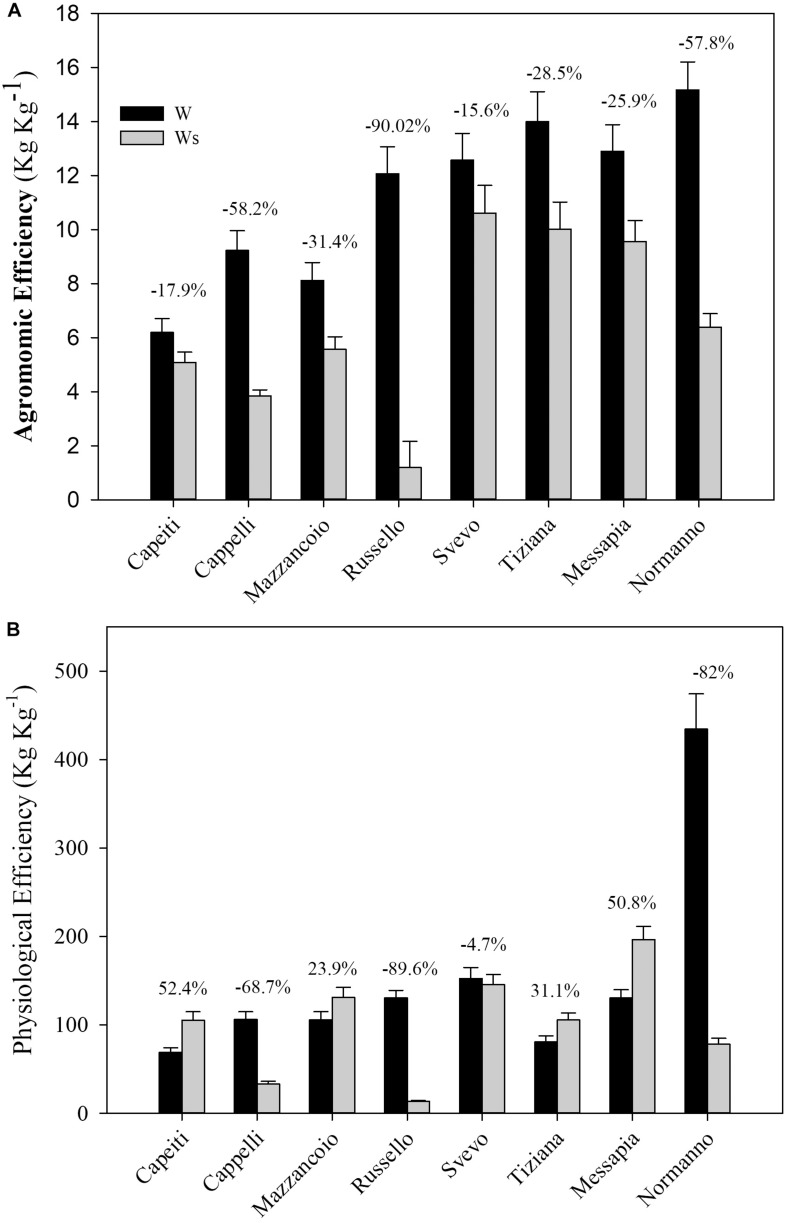
Agronomic **(A)** and physiological **(B)** efficiency of eight durum wheat genotypes exposed to two water regimes in the field. Values are means (*n* = 5) ± SE.

The Ws affected AE in all the genotypes, ranging from Russello exhibiting the most marked reduction (90%) to Cappelli (76%), Capeiti (59%), and Svevo (15.6%) (Figure 2A).

The PE appeared significantly increased in Capeiti (52.4%), Messapia (50.7%), Tiziana (31.1%), and Mazzancoio (23.9%). In contrast, Cappelli, Normanno, and Russello exhibited a consistent PE reduction by 68.7, 82, and 89.6%, respectively (Figure 2B).

#### Water Use Efficiency

**FIGURE 3:**
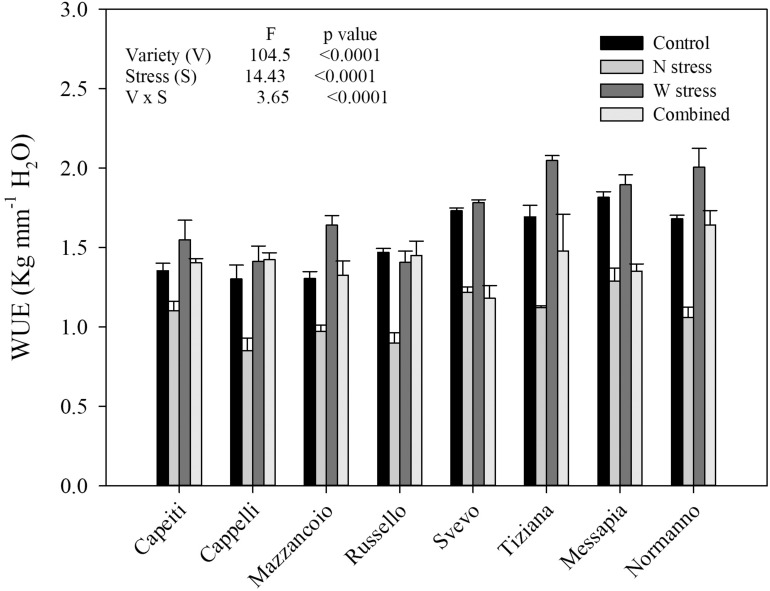
Water use efficiency of eight durum wheat varieties under water and nitrogen stress alone or in combination in the field experiment. Values are means (*n* = 5) ± SE.

Water use efficiency was estimated in all the varieties and at both stress conditions (Figure 3). Analysis of variance underlined the high significance (*p* < 0.001) of all the main factors (variety and both stress) as well as their interaction (*p* < 0.001). At the control, WUE ranged from 1.30 (Cappelli) to 1.81 (Messapia), with a 1.54 average evidencing significant differences among the varieties. Moreover, all the varieties exhibited a WUE reduction (average 1.06) under N stress, albeit ranging from Capeiti (−18%) to Russello (−38%). As expected, under W stress, WUE tended to increase in all the varieties except for Russello, which showed a limited and not significant reduction compared to the control. Finally, the combined stress determined contrasting responses among the varieties. Cappelli and Russello restored WUE at the control level under both stresses (Figure 3).

#### Heritability

To determine the percentage of explained variance due to genetics, environment (as W and N stress), and their interaction, global ANOVA was performed for all the traits (Supplementary Figure S2). The higher rate of genetic variation was registered for heading days (96.7%) and plant height (89%), followed by HI (42.8%), NUtE (30.5%), and dry weight at AN (30.4%), whereas the smallest rate of genetic contribution was found in NUpE (0.2%) (Supplementary Figure S2).

#### Stress Tolerance Index Among Genotypes

**FIGURE 4:**
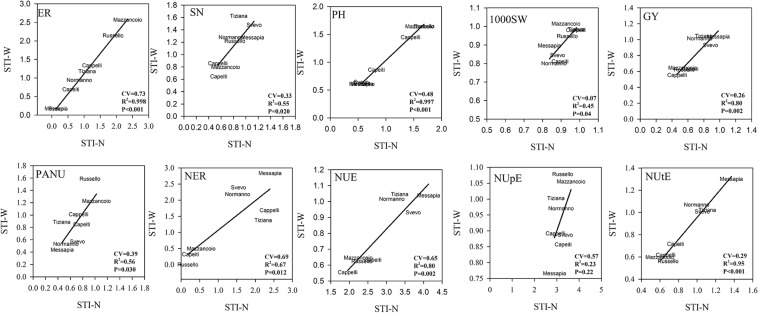
Biplot of stress tolerance index (STI) for N and water stress for each trait. Pearson’s correlation was calculated, and *R*^2^ as well as their significance are reported. Coefficient of variation was also reported (for STI calculation, see section “Materials and Methods”).

The biplot analysis using STI-W *vs*. STI-N allowed us to describe the different performances of all the varieties and to explain their different tolerance to W, N, and combined stress. In detail, the localization in the dials of each variety, by plotting the two indices, indicated the tolerance degree to each stress. The CV and the correlation (*R*^2^), calculated for each trait, described the phenotypic variability and the relationship between the responses of each variety to both stresses (Figure 4). The earliness showed the highest CV (=0.73) and a significant correlation (*R*^2^ = 0.998) among the traits, resulting in the distribution along a diagonal line in the graph, from Mazzancoio to Messapia and Svevo, the most tolerant varieties to both stresses. The lowest CV accompanied by a significant *R*^2^ between stress was recorded for 1000SW, indicating a marked similarity for this trait in all the varieties. The SN displayed low CV (0.33) and *R*^2^ (0.55), with a rank of tolerance to both stresses from Tiziana and Svevo (tolerant) to Capeiti, Cappelli, and Mazzancoio (sensitive) (Figure 4).

More interestingly, a relatively high correlation (*R*^2^ = 0.80) associated with a lower CV (0.26) was evident for GY, where two clusters were well distinguishable: a tolerant cluster including Svevo, Messapia, Normanno, and Tiziana with higher values of both indices (tolerant) and a sensitive cluster comprising Cappelli, Capeiti, Mazzancoio, and Russello (Figure 4). A low CV (0.39) together with a significant *R*^2^ was instead recorded for PANU, where Russello and Mazzancoio showed higher tolerance to both stresses compared to Messapia, Normanno, and Svevo, the more sensitive ones. Russello, Capeiti, and Mazzancoio showed lower values in both indices when considering NER (CV = 0.69 and *R*^2^ = 0.67), unlike Messapia (Figure 4).

Finally, two distinct clusters for NUE (CV = 0.65 and *R*^2^ = 0.80) and NUtE (CV = 0.29 and *R*^2^ = 0.95) were distinguishable, including the modern varieties (Messapia, Tiziana, Normanno, and Svevo), which were more efficient in N use and utilization at both stresses compared to the oldest ones. In contrast, all the varieties showed similar responses to N stress except for the NUpE component which was not able to determine a clustering between groups of genotypes due to a very low correlation between stresses (*R*^2^ = 0.23) (Figure 4).

#### Pearson’s Correlation and Principal Component Analysis

**FIGURE 5:**
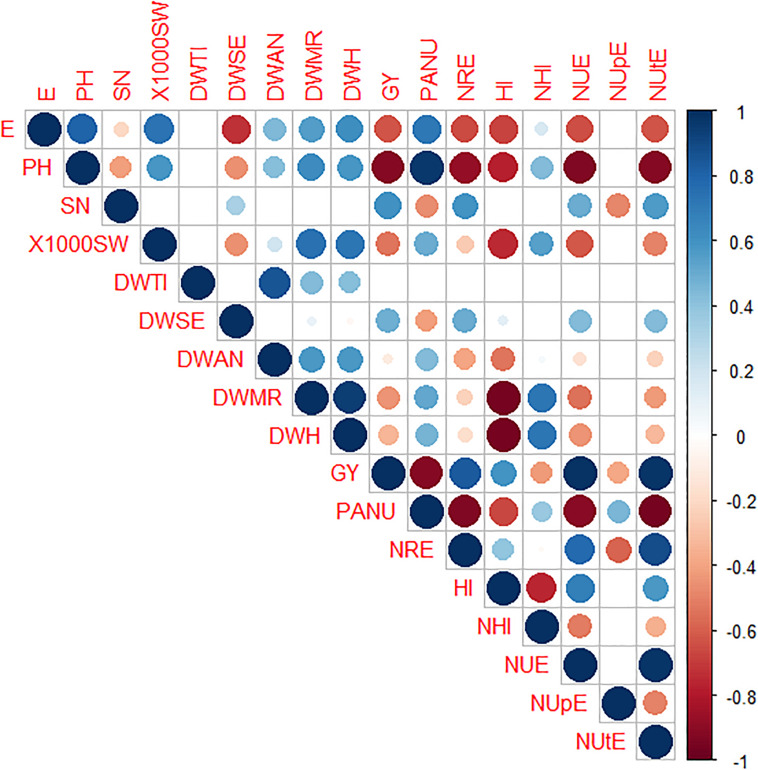
CorPlot of Pearson’s correlation among morpho-physiological traits in eight durum wheat genotypes. Positive and negative correlations are displayed in blue and red, respectively. The color intensity and the circle size are proportional to the correlation coefficients. The absence of circles in the squares indicates values with significance of *p* > 0.05. DWTI, dry weight tillering; DWSE, dry weight stem extension; DWA, dry weight at anthesis; DWMR, dry weight at milk ripening; DWH, dry weight harvest; E, earliness; PH, plant height; 1000SW, thousand seed weight; SN, spike number; GY, grain yield; HI, harvest index; NHI, nitrogen harvest index; PANU, post-anthesis nitrogen uptake; NRE, nitrogen remobilization efficiency; NUE, nitrogen use efficiency; NUpE, nitrogen uptake efficiency; NUtE, nitrogen utilization efficiency.

Pearson’s correlation between traits showed the highest positive values between NUE and NUtE (0.97), NUE and GY (0.97), and NUtE and GY (0.96) as expected, PANU and PH (0.96) as well as dry weight at milk ripening (DWMR) and at harvesting (DWH) (0.94). In contrast, the highest negative correlations were detected for DWMR and DWH *vs*. HI (−0.97 and −0.96, respectively), NUE, NUtE, NRE, and GY *vs*. PH (−0.92, 0.92, −0.91, and −0.91, respectively). Similar negative correlation values were also observed between NUE, NUtE, NRE, and GY *vs*. PANU (Figure 5).

The PCA was performed by computing crop yield main components as well as physiological traits. The PC1 and PC2 explained 51.2 and 21.4% of the variance, respectively (Supplementary Figure S3). Two clusters, named group 1 (G1) and group 2 (G2), were identified as previously reported for some traits in the biplot (see Figure 4). The G1 included the modern varieties Messapia, Tiziana, Svevo, and Normanno, mainly based on NUE, NUtE, and NRE, whereas Russello, Mazzancoio, Cappelli, and Capeiti were included in G2, showing similar NUpE and PANU (Supplementary Figure S3).

#### Characterization of Two Different Groups of Genotypes

**FIGURE 6:**
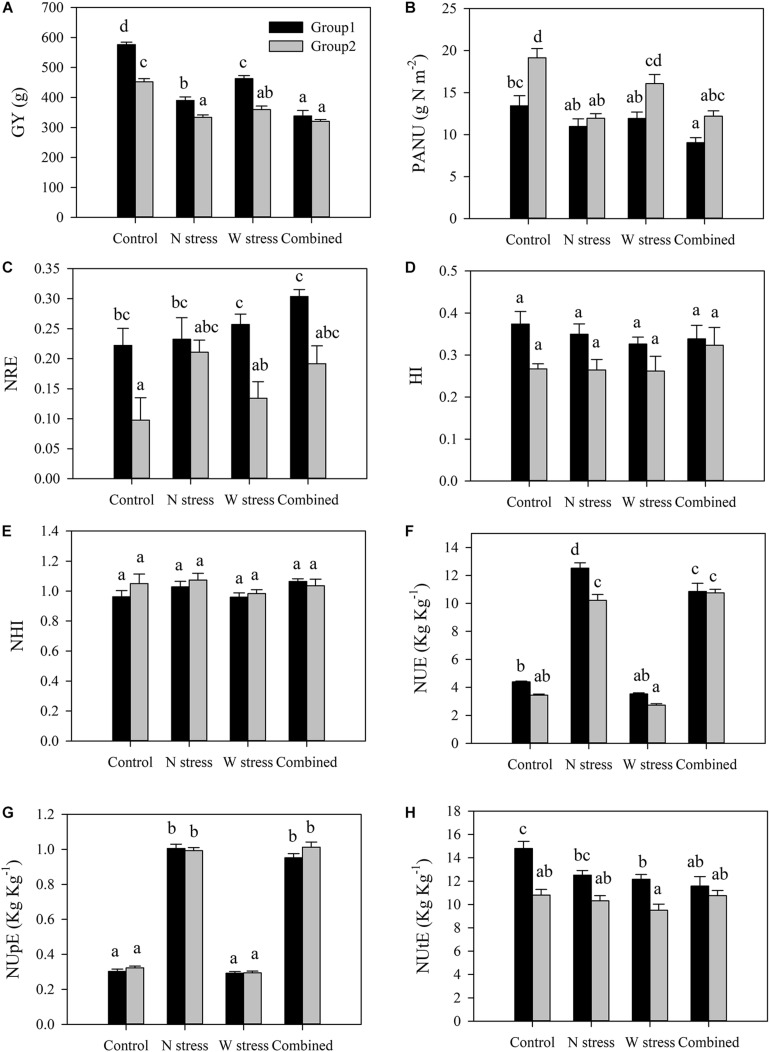
Yield and N-related physiological traits of two groups of genotypes: group 1 (Messapia, Tiziana, Svevo, and Normanno) and group 2 (Russello, Mazzancoio, Cappelli, and Capeiti). Values are means (*n* = 5) ± SE. Different letters indicate means that differ significantly according to Tukey’s honest significant difference test at *p* < 0.05. GY, grain yield **(A)**; PANU, post-anthesis nitrogen uptake **(B)**; NRE, nitrogen remobilization efficiency **(C)**; HI, harvest index **(D)**; NHI, nitrogen harvest index **(E)**; NUE, nitrogen use efficiency **(F)**; NUpE, nitrogen uptake efficiency **(G)**; NUtE, nitrogen utilization efficiency **(H)**.

To outline the different responses to stress, alone or in combination, of G1 and G2, previously identified by biplot (Figure 4) and confirmed by PCA (Supplementary Figure S3), GY and several physiological traits were compared (Figure 6). G1 included the modern durum wheat varieties Messapia, Tiziana, Svevo, and Normanno, while G2 included the old landraces Russello and Mazzancoio from Sicilian and Calabria, respectively, and the old varieties Cappelli and Capeiti. Regardless of stress exposure, GY was significantly higher in G1 than in G2, as expected. Nevertheless, N, W, and combined stresses significantly reduced GY in G1 by 32, 19, and 42%, respectively, compared to the control, whereas a lower reduction was observed in G2 (26, 20, and 29%, respectively) (Figure 6A). Interestingly, under control as well as W stress, a higher PANU was observed in G2 compared to G1 (Figure 6B). In contrast, under the same conditions, G1 exhibited a higher NRE compared to G2 (Figure 6C). G1 and G2 did not show any significant differences in HI and NHI (Figures 6D,E). As expected, both groups showed a significantly higher NUE under N alone and combined stress, although G1 exhibited a higher NUE compared to G2 under N stress. However, these differences between groups disappeared under combined stress (Figure 6F). Significant differences among treatments were observed for NUpE but not between groups, as expected, by biplot analysis (Figure 6G). Finally, G1 showed a higher NUtE in the control and under water stress when compared to G2. More interestingly, water stress significantly reduced NUtE in G1, whereas G2 did not show any significant differences at all the treatments compared to the control (Figure 6H).

#### Usage Index Over the Life Cycle

**FIGURE 7:**
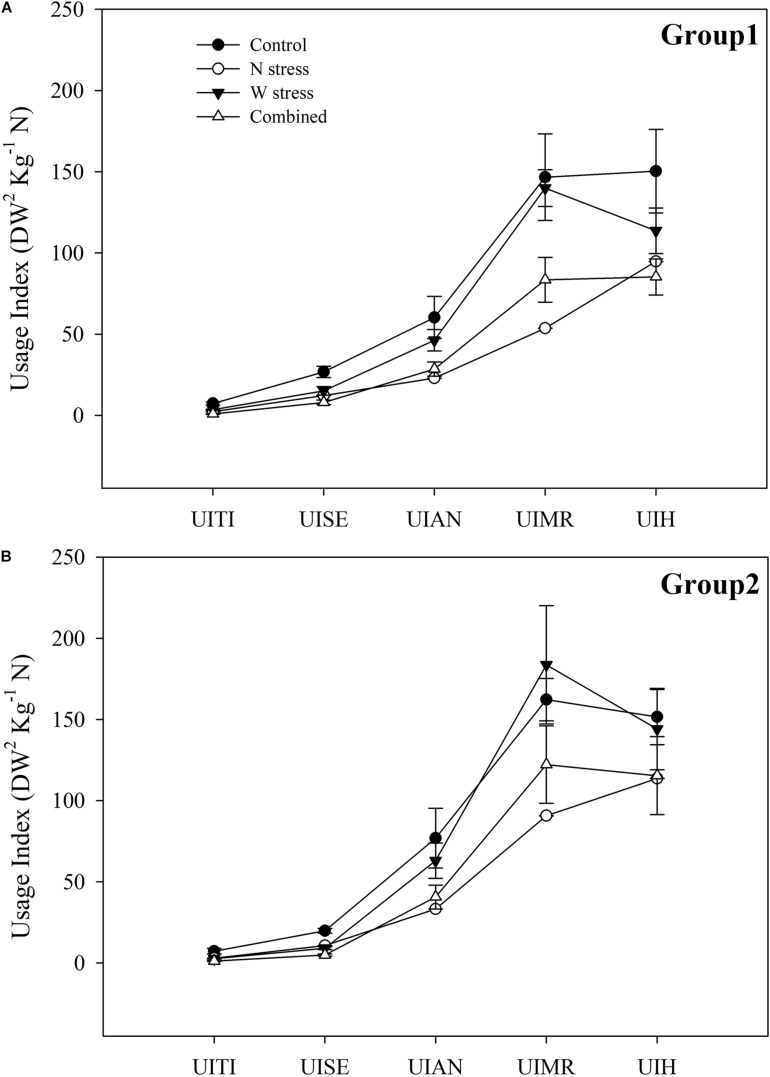
Usage index of nitrogen during phenological stages of durum wheat genotypes. Group 1: Messapia, Tiziana, Svevo, and Normanno **(A)** and group 2: Russello, Mazzancoio, Cappelli, and Capeiti **(B)** calculated according to Siddiqi and Glass (1981). UITI, usage index at tillering; UISE, usage index stem extension; UIAN, usage index at anthesis; UIMR, usage index at milk ripening; UIH, usage index at harvest.

To determine the key stages of wheat life cycle affected by stress, alone or in combination, and to visualize the different responses between G1 and G2, the UI, as dry biomass yielded for available N, was calculated. The UI allowed us to evaluate either the increase in biomass or, concurrently, the N accumulation amounts at each stage of the life cycle (Figure 7).

In detail, both groups displayed a similar pattern alongside the life cycle at the control (Figure 7). Under N stress, G1 exhibited a more marked UI reduction compared to the control at all stages and by 69, 54, 61, 63, and 37% at tillering (UITI), stem extension (UISE), anthesis (UIAN), milk ripening (UIMR), and harvesting (UIHA), respectively (Figure 7A). In contrast, G2 showed a less marked UI reduction by 59, 45, 56, 44, and 24% compared to the control, especially at MR (63 *vs*. 44% and G1 *vs*. G2, respectively). G1 and G2 showed a weak UI reduction compared to the control when water stress was applied, although G2, starting from anthesis, exhibited a lower UI reduction or even an increase at MR (13%, with respect to the control) and at harvest (Figure 7B). Finally, a similar trend between the groups was observed when both stresses were applied, showing a marked UI reduction compared to the control at TI, SE, and AN. Interestingly, at MR and H, G1 showed a significantly higher UI reduction by 43 and 43% compared to 24 and 23% of G2, respectively. Thus, G2 showed a significantly higher tolerance to combined stress compared to G1 (Figure 7).

#### Gene Expression

**FIGURE 8:**
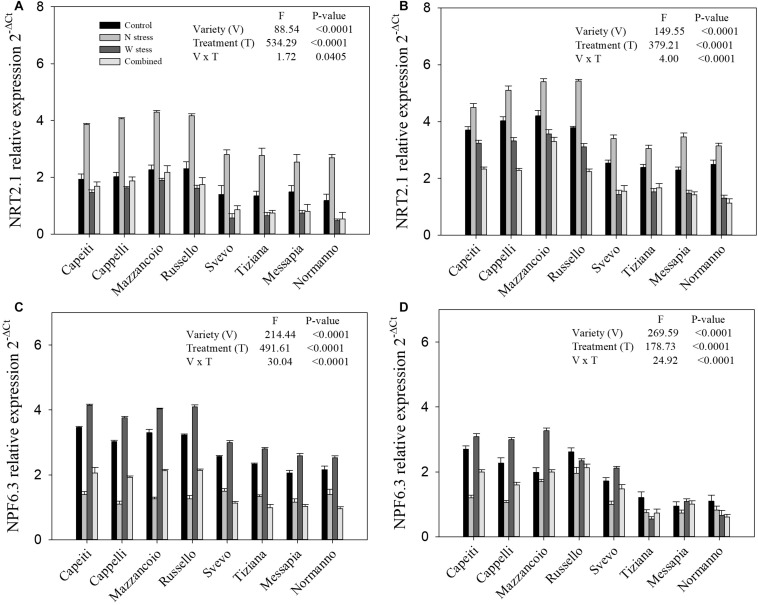
Expression of genes relative to nitrate transporter in durum wheat root. *NRT2.1* and *NPF6.3* relative expression at stem elongation **(A,C)** and milk ripening **(B,D)** are shown.

**FIGURE 9:**
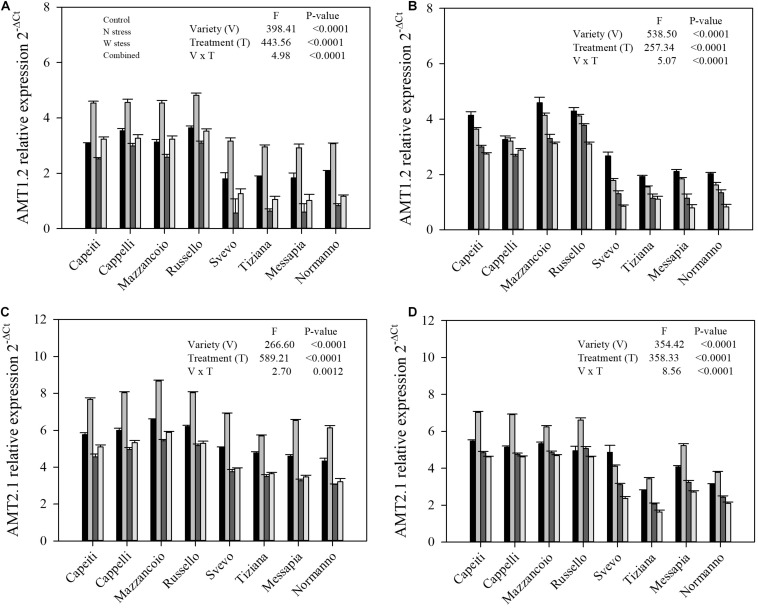
Expression of gene relative to ammonium transporter in durum wheat root. *AMT1.2* relative expression at stem elongation **(A)** and milk ripening **(B)** are shown; *AMT2.1* relative expression at stem elongation **(C)** and milk ripening **(D)** are shown.

At SE and MR, the gene expression profiles for nitrate (*NRT2.1* and *NPF6.3*) and ammonium (*AMT1.2* and *AMT2.1*) transporters of wheat genotypes exposed to N and water stress, alone or in combination, were evaluated by RT-PCR (Figures 8, 9). The *NRT2.1* expression displayed significant differences in varieties, stress applied, and their interaction at both phenological stages (Figures 8A,B). At the control, the ancient varieties (Mazzancoio, Cappelli, Capeiti, and Russello) exhibited a higher expression level compared to the modern ones (Svevo, Messapia, Normanno, and Tiziana) at both stages (Figures 8A,B). Under N stress, a significant increase in *NRT2.1* transcript abundance was detected in all the varieties at SE stage, ranging from 70% (Messapia) to 127% (Normanno), compared to the control (Figure 8A). Besides this, under water and combined stress, a moderate reduction of gene expression was observed in all the varieties, albeit with some differences among varieties. In detail, under combined stress, *NRT2.1* expression reduction compared to the control was observed, ranging from 16% (Mazzancoio) to 59% (Normanno), with the best performances in Mazzancoio, Cappelli, and Capeiti (Figure 8A).

Under N stress, an expected increase of *NRT2.1* expression in all the varieties compared to the control was observed at MR stage, ranging from 21% (Capeiti) to 50.7% (Messapia). In contrast, a reduction of transcript abundance was observed under water as well as combined stress. Mazzancoio and Capeiti confirmed their best performance with a limited reduction in gene expression, whereas Normanno showed the worst one (Figure 8B).

At the control, the dual-affinity transporter *NPF6.3* showed a higher expression in the ancient varieties than the modern ones at both stages (SE and MR), as already observed for *NRT2.1* (Figures 8C,D). At SE, the *NPF6.3* transcript abundances were reduced in all the genotypes compared to the control under both N and combined stress; in contrast, water stress moderately increased its gene expression (from 16 to 26% by Svevo and Russello, respectively) (Figure 8C). At MR, similar trends in *NPF6.3* gene expression were detected in Mazzancoio, Cappelli, Capeiti, and Svevo when compared to SE, whereas the other varieties did not show significant differences among treatments (Figure 8D).

Furthermore, gene expression levels related to ammonium transporters (*AMT1.2* and *AMT2.1*) at SE and MR were reported (Figure 9). Significant differences (*p* < 0.0001) related to variety, stress, and their interaction for both genes and stages were observed, as well as for nitrate transporters (Figures 9A–D). At SE stage, *AMT1.2* expression level increased in all the genotypes under N stress compared to the control (from 29 to 75% by Cappelli and Svevo, respectively). Water as well as combined stress reduced the *AMT1.2* transcript abundances in the modern varieties, whereas the ancient ones did not show any differences compared to the control (Figure 9A). At MR stage, the modern varieties highlighted a lower *AMT1.2* expression at the control, whereas all the stress reduced its transcript abundances in all the varieties (Figure 9B). Finally, N stress increased the *AMT2.1* expression in all the genotypes, although with differences among varieties, while W and combined stress significantly reduced its expression when compared to the control only in the modern varieties at both stages (Figures 9C,D).

## Discussion

A high N requirement is necessary for plant growth, crop yield, and quality, thus the optimization of N fertilizer management along with the adoption of high NUE wheat varieties is the most promising strategy to maximize crop yield and efficiently safeguard the environment (Arregui and Quemada, 2008; Zörb et al., 2018). Besides N, water availability is another of the major limiting factors for wheat yield (Austin, 2011), and unfortunately rainfall patterns in many world regions, including the Mediterranean Basin, are becoming unpredictable (Monjon and Martin-Vide, 2016). Therefore, the selection of resilient crop varieties has become one of the main objectives to tackle abiotic stress. In the present study, ancient and modern durum wheat varieties were compared, taking into account the key role of N metabolism-related genes in the transition from landraces to modern varieties in durum wheat as recently reported (Gioia et al., 2015). In detail, NUE and related physiological traits among genotypes exposed to water and nitrogen stress, alone or in combination, were assessed to identify genotypes able to maintain a high NUE.

The variability for NUE and its components into the tetraploid wheat panel was firstly evaluated in the growth chamber pot experiment, which provided a controlled environmental condition to better analyze abiotic stress effects with lower costs (Rebetzke et al., 2014; Yadav et al., 2019). Considering the total biomass increase, this experiment allowed us to identify a significant variability in NUE and its components among genotypes. Although the variety × stress (V × S) interaction did not exhibit significant differences in NUE and its components, either NUE or NUtE was characterized by a marked reduction under water stress, unlike the N-limiting condition. These first results chiefly indicated that our panel is suitable for field trials based on NUE variability, at a greater or lesser extent, among genotypes under water stress.

Then, the genotype response to water and N stress, alone or in combination, was compared in field conditions. Firstly, we outlined that the arrangement of aluminum channels in the soil made it possible to simulate water stress due to the significant differences on many traits between rainfed and water-stressed main plots. Moreover, global ANOVA indicated a high contribution of genetic contribution to the phenotypic variation in some key parameters such as NUtE, HI, PANU, and biomass in our genotype panel. Interestingly, some genotypes, e.g., the old landrace from Calabria (Mazzancoio) and the old variety Cappelli, accumulated a higher biomass alongside different phenological stages during their life cycle. Dry matter increase at harvest may be considered as an achievable and feasible strategy in breeding programs (Aparicio et al., 2002; Hawkesford, 2017). In detail, Mazzancoio exhibited higher biomass from tillering to anthesis, thereby demonstrating a potential tolerance to both stresses. Interestingly, the increase in biomass before anthesis was considered as a pivotal response for improving crop yield in cereals (Slafer et al., 2005; Estrada-Campuzano et al., 2012). So far, the identification of some NUE-related traits in these genotypes (Mazzancoio and Cappelli) may be useful for breeding programs to improve NUE performance under multiple abiotic stresses (N plus water). Indeed the increase of above-ground dry matter under abiotic stress was frequently correlated to crop yield (Miralles and Slafer, 2007; Reynolds et al., 2007; Estrada-Campuzano et al., 2012). Among the physiological parameters, HI did not exhibit any significant differences under N and water stress, and these results appeared to be in agreement with those of Mahjourimajd et al. (2016), demonstrating that a different N rate supply did not influence HI in an Australian wheat mapping population.

Post-anthesis nitrogen uptake is an important parameter for identifying higher-yield wheat variety (Monaghan et al., 2001), which allowed us to distinguish the contrasting behavior between ancient and modern varieties under normal and limited N supply. Indeed the ancient varieties (Russello, Mazzancoio, Cappelli, and Capeiti) exhibited higher PANU values compared to the modern ones. This contrasting behavior between ancient and modern varieties was also supported by the main N uptake-related gene expression analysis (*NRT2.1*, *NPF6.3*, *AMT2.1*, and *AMT1.2*). Indeed the best performing genotypes for PANU showed the highest transcript abundances for *NRT2.1* and *AMT2.1*, at control and stress conditions, during both pre- and post-anthesis stages. In wheat, a correlation between N transporter expression level and NUE, with critical differences during grain filling and other NUE-related processes, was recently highlighted (Hawkesford, 2017). More recently, Guo et al. (2019), comparing three wheat cultivars with different N efficiency, demonstrated that the more efficient cultivars exhibited a higher expression of nitrate transporters compared to the inefficient ones. It is noteworthy that these differences were more marked in pre-filling stages, according to our results, in which gene expression was performed at SE and MR stages. In contrast, as previously observed by Duan et al. (2016), the ammonium transporters were markedly induced by water stress in the vegetative stage. However, further studies are needed to better understand the relative contribution of NRT and AMT transporters.

Furthermore, a significant correlation was previously observed between PANU and grain protein deviation (GPD), which indicated the deviation between grain yield and grain protein content by regression, appearing under a stout genetic control (Monaghan et al., 2001; Bogard et al., 2010; Taulemesse et al., 2016). These latter authors characterized a bread wheat genotype with positive GPD supported by an increased ability to uptake nitrogen (N) at maturity, regardless of N amount taken up before flowering (Taulemesse et al., 2016). Their results were sustained by a higher *NRT2.1* expression alongside flowering as we have observed in the ancient varieties. The PANU-correlated traits useful to select genotypes with higher grain protein content could represent as one of the main targets in wheat breeding programs (Branlard et al., 2001; Oury et al., 2010).

However, in addition to NUE and its components, other measures should be taken into account when a genotype collection is assessed for improving this complex trait (Moll et al., 1982; Wu et al., 2011). The AE comparing two diverse watered plots as well as N treatments evidenced variability among genotypes, in which the modern varieties showed higher AE under optimal water availability. In contrast, under water stress, a lower AE reduction in ancient varieties was observed, and this behavior could be due to the selection strategy adopted for the constitution of modern varieties (Taranto et al., 2020). According to AE results, the PE evidenced a similar contrasting behavior between ancient and modern genotypes, suggesting the greater ability of the ancient varieties to withstand water stress than the modern ones.

Moreover, to better understand the genotype responses to stress, alone or in combination, the tolerance index to both stresses (STI-W and STI-N) were estimated. A clustering of different behaviors between ancient and modern varieties was observed for many traits including AE and PE data. In detail, modern varieties showed higher tolerance to both stresses than the ancient ones for NUE (CV = 0.65, *R*^2^ = 0.80) and NUtE (CV = 0.29, *R*^2^ = 0.95). These results confirmed some recent evidence on the possible indirect selection by breeders of wheat varieties with improved NUE, as a result of choosing higher-yielding varieties (Cormier et al., 2013; Taranto et al., 2020). In particular, Taranto et al. (2020) outlined the selection impact on the Italian durum wheat genetic diversity where the analysis of diversity patterns resulted in the detection of major QTL that could define the differences between ancient and modern varieties. Interestingly, these QTL affected plant height, earliness, and grain quality, many of which were localized in genomic regions where N metabolism-related genes were mapped (Taranto et al., 2020). In agreement, our results highlighted that higher NUE varieties presented all these traits and were included in the modern varieties namedgroup 1.

In contrast, ancient varieties exhibited higher values of both STI compared to the modern varieties for PANU, a trait useful for pyramiding QTL to improve NUE.

Overall, the significant correlation between STI-N and STI-W for several traits confirmed that tolerance mechanisms to both stresses could be pyramided in such genotypes, and these results were supported by the correlation between NUE and WUE either at low or high N supply. It is well known that N and W use efficiency evolved from diploid to hexaploid in a similar way (Li et al., 2003), and some genes linked to both NUE and WUE have just been mapped on the same chromosomes (Zhou et al., 2006; An et al., 2006).

Furthermore, PCA confirmed two distinct clusters of genotypes according to their year of constitution, although the older Capeiti appeared slightly different from Cappelli, Russello, and Mazzancoio, according to its pedigree (De Cillis, 1942; Fiore et al., 2019).

Finally, the UI analysis along the durum wheat life cycle pointed out well the ability of ancient varieties to accumulate more biomass in pre- and post-anthesis compared to the modern ones under W stress. It is noteworthy that the highest UI at milk ripening outlined by G2 under both stresses, alone and in combination, resulted to be of very high interest, confirming the higher tolerance of the durum wheat ancient landrace/varieties to abiotic stress.

## Conclusion

The old landraces from Calabria and Sicily (Mazzancoio and Russello) as well as the ancient genotypes (Cappelli and Capeiti) tend to promote vegetative growth, showing a higher tolerance to both stresses in both growth chamber and field experiments, while grain filling efficiency was higher in the modern ones. Thus, our data demonstrated that the selection of varieties with high N use efficiency led to lowered tolerance to other abiotic stress. In this respect, ancient varieties could represent suitable genetic resources useful in durum wheat breeding programs for selecting genotypes adaptable to a more sustainable cropping system, characterized by low rainfall and N fertilizer input, in the Mediterranean environment.

## Data Availability Statement

The raw data supporting the conclusions of this article will be made available by the authors, without undue reservation.

## Author Contributions

MB, GP, MM, and FS planned the experiments. GB and AL carried out the growth chamber experiment and extracted the RNAs. GP, MB, GB, and MM carried out the field experiment. GP, MM, MA, FS, and AL analyzed the data. MB, MA, FS, GP, and AL were involved in drafting the manuscript. MM, MA, FS, and AL critically discussed and finalized the manuscript. All the authors read and approved the final version of the manuscript.

## Conflict of Interest

The authors declare that the research was conducted in the absence of any commercial or financial relationships that could be construed as a potential conflict of interest.

## Supplementary Material

Supplementary Figure 1 Monthly rainfall (bars), mean, maximum (circles), and minimum temperatures (triangles) at the experimental station in the season 2014/2015.

Supplementary Figure 2 Phenotypic variance partitioning of morpho-physiological durum wheat traits under different N and water availability. DWTI, dry weight tillering; DWSE, dry weight stem extension; DWAN, dry weight at anthesis; DWMR, dry weight at milk ripening; DWH, dry weight harvest; E, earliness; PH, plant height; 1000SW, thousand seed weight; SN, spike number; GY, grain yield; HI, harvest index; NHI, nitrogen harvest index; PANU, post-anthesis nitrogen uptake; NRE, nitrogen remobilization efficiency; NUE, nitrogen use efficiency; NUpE, nitrogen uptake efficiency; NUtE, nitrogen utilization efficiency.

Supplementary Figure 3 Principal component analysis of the wheat varieties used based on bio-morphological parameters. DWTI, dry weight tillering; DWSE, dry weight stem extension; DWAN, dry weight at anthesis; DWMR, dry weight at milk ripening; DWH, dry weight harvest; E, earliness; PH, plant height; 1000SW, thousand seed weight; SN, spike number; GY, grain yield; HI, harvest index; NHI, nitrogen harvest index; PANU, post-anthesis nitrogen uptake; NRE, nitrogen remobilization efficiency; NUE, nitrogen use efficiency; NUpE, nitrogen uptake efficiency; NUtE, nitrogen utilization efficiency.

Supplementary Figure 4 Pearson’s correlation between water use efficiency and nitrogen use efficiency at low and high N level for each genotype.

Supplementary Table 1 List of genotypes employed in the present study and pedigree origin.

Supplementary Table 2 Forward and reverse primers for gene amplification in real-time qPCR.
