# Supplementary material for: Nitrogen Use Efficiency in Durum Wheat Under Different Nitrogen and Water Regimes in the Mediterranean Basin
Source: Front Plant Sci. 2021 Feb 10;11:607226. doi: 10.3389/fpls.2020.607226 (PMC7902889; doi:10.3389/fpls.2020.607226)
Supplement: Supplementary Figure 1 — Monthly rainfall (bars), mean, maximum (circles), and minimum temperatures (triangles) at the experimental station in the season 2014/2015. [file Data_Sheet_1.PDF]

Supplementary Materials

Supplementary Table 1. Origin and pedigree, morphologic and physiological traits of durum wheat genotypes employed.

Variety	Pedigree	Responsible for conservation	Plant height	Biological cycle	Tillering
Cappelli	Landrace Jeanh Rhetifah	Strampelli (1923)	High	Late	Limited
Capeiti 8	Eiti 6 x Cappelli	Casale (1955)	Average-high	Medium-late	Limited
Russello	Landrace Sez. Europea	De Cillis (1964)	High	Late	Limited
Mazzancoio	Collected in Calabria	UNIRC (1997)	High	Late	Limited
Messapia	(Mex x Crane "S") x Tito	Ist. M.G. - Bari (1985)	Average	Early	Medium
Svevo	Sel. Cimmyt x Zenit sib	S.P.S.-Bologna (1996)	Average	Early	Good
Normanno	Sleto x F22 x L35	S.P.S.-Bologna (1996)	Average	Medium	Medium
Tiziana	Peleo x Neodur	CO.NA.SE (2001)	Average	Medium-late	Good

Supplementary Table 2. Forward and reverse primers for gene amplification in real time qPCR.

Gene	Locus	Primer sequences
NPF6.3	AY587265.1	F: CACAGCGAATAGGGATTGGT<br>R: CGCCTAGCAGGAAGTACTGG
NRT2.1	AF288688.1	F: GTGGTGCCACACAACTCATC<br>R: TTCTGGAGACTCGCAAGGTT
AMT1.2	AY525638.1	F: CGGCTTCGACTACAGCTTCT<br>R: AGTGGGACACCACAGGGTAG
AMT2.1	AY428038.1	F: AGCCGAACCTCTGCAATCTA<br>R: TGACGACGCAGATAATGGAC
18S	AB778770.1	F: CAACGGATATCTCGGCTCTC<br>R: TTGCGTTCAAAGACTCGATG

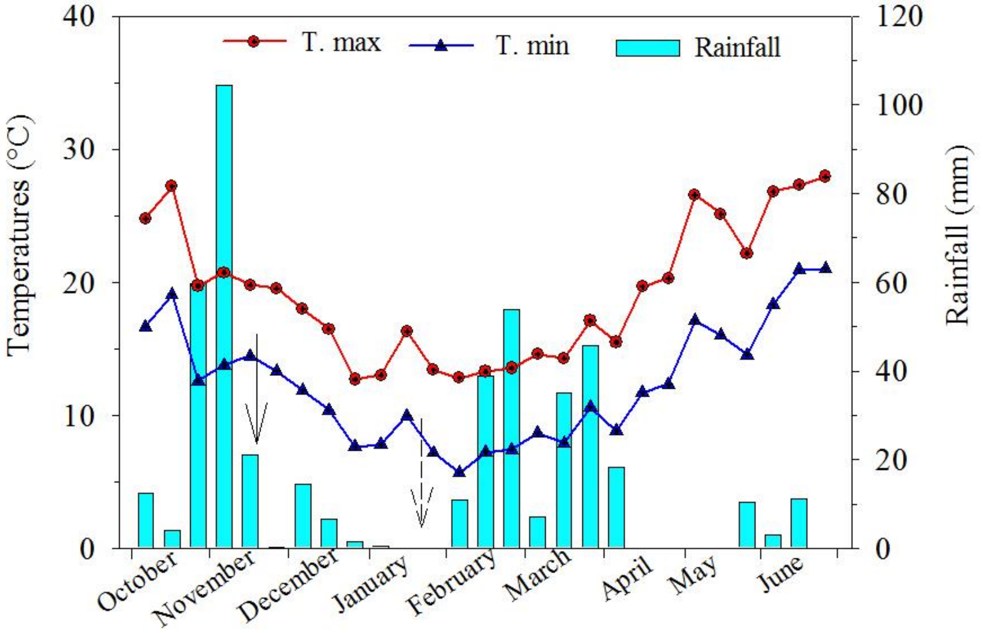

Supplementary Figure 1. Monthly rainfall (bars), mean, maximum (circle symbols) and minimum temperatures (triangle symbols) at experimental station in the season 2014/2015.

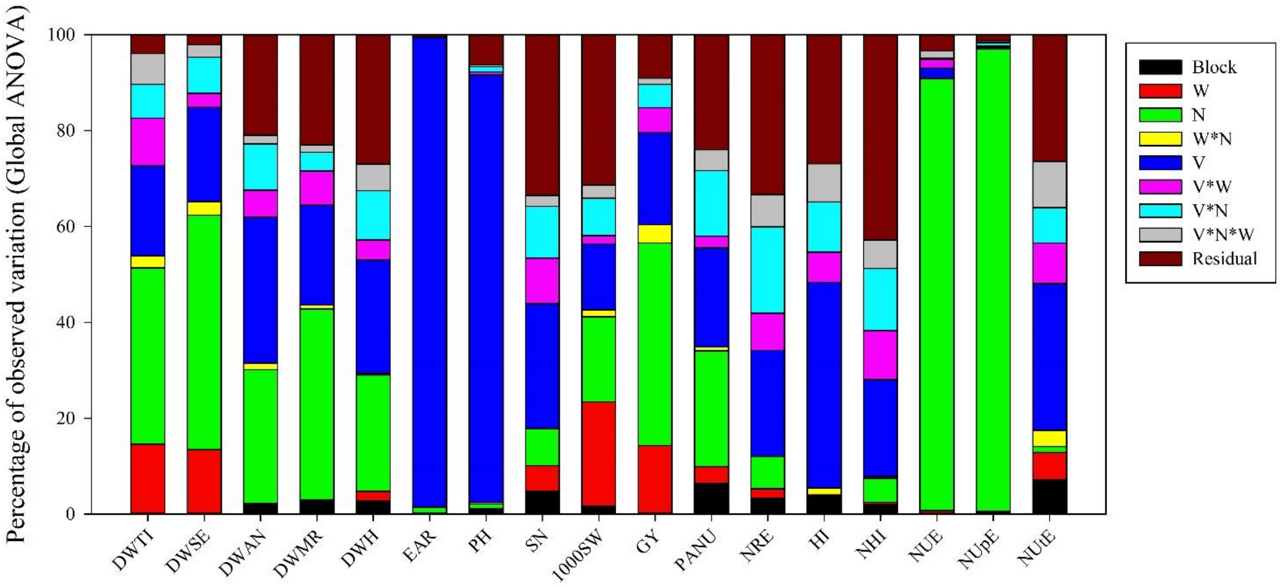

Supplementary Figure 2. Phenotypic variance partitioning of morpho-physiological durum wheat traits under different N and W availability. DWTI, Dry Weight Tillering; DWSE, Dry Weight Stem extension; DWAN, Dry Weight at Anthesis; DWMR, Dry Weight at Milk Ripening; DWH, Dry Weight Harvest; E, Earliness; PH, Plant Heigh;1000SW, Thousand Seed Weight; SN, Spike number; GY, Grain Yield. HI, Harvest Index; NHI, Nitrogen Harvest Index; PANU, Post-Anthesis Nitrogen Uptake; NRE, Nitrogen Remobilization Efficiency; NUE, Nitrogen Use Efficiency; NUpE, Nitrogen Uptake Efficiency; NUtE, Nitrogen Utilization Efficiency

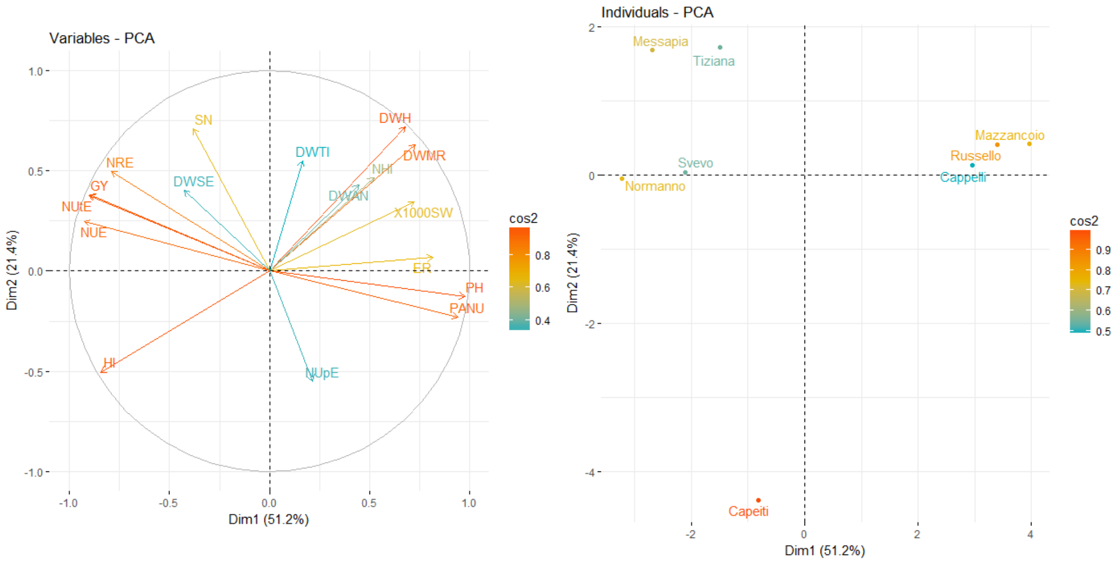

Supplementary Figure 3. Principal component analysis of the wheat varieties used based on the bio-morphological parameters. DWTI, Dry Weight Tillering; DWSE, Dry Weight Stem extension; DWAN, Dry Weight at Anthesis; DWMR, Dry Weight at Milk Ripening; DWH, Dry Weight Harvest; E, Earliness; PH, Plant Heigh;1000SW, Thousand Seed Weight; SN, Spike number; GY, Grain Yield. HI, Harvest Index; NHI, Nitrogen Harvest Index; PANU, Post-Anthesis Nitrogen Uptake; NRE, Nitrogen Remobilization Efficiency; NUE, Nitrogen Use Efficiency; NUpE, Nitrogen Uptake Efficiency; NUtE, Nitrogen Utilization Efficiency

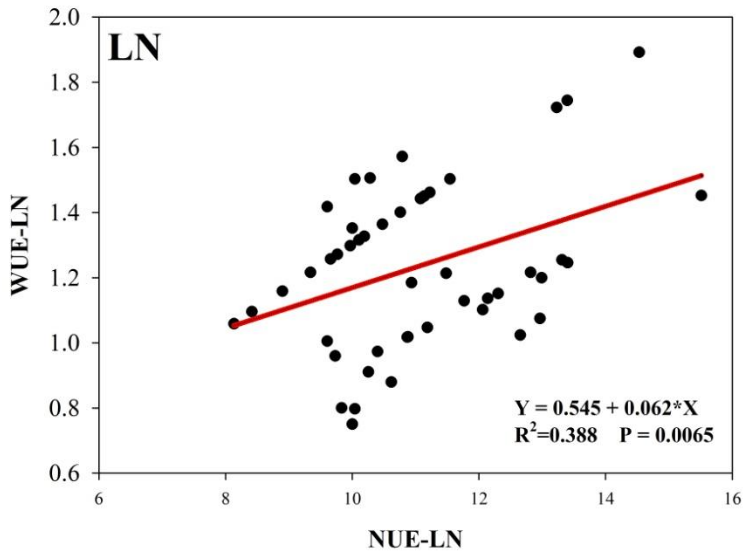

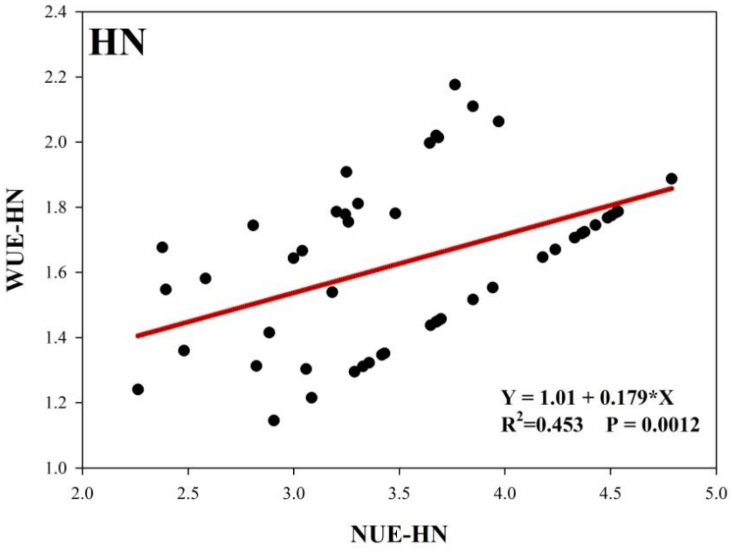

Supplementary Figure 4. Pearson's correlation between WUE and NUE at low and high N level for each genotype.
